# Evolutionary anatomies of positions and types of disease-associated and neutral amino acid mutations in the human genome

**DOI:** 10.1186/1471-2164-7-306

**Published:** 2006-12-05

**Authors:** Sankar Subramanian, Sudhir Kumar

**Affiliations:** 1 Center for Evolutionary Functional Genomics, The Biodesign Institute and School of Life Sciences, Arizona State University, Tempe, Arizona 85287-5301, USA; 2 Allan Wilson Centre for Molecular Ecology and Evolution, Institute of Molecular Biosciences, Massey University, Private Bag 102904, Auckland, New Zealand

## Abstract

**Background:**

Amino acid mutations in a large number of human proteins are known to be associated with heritable genetic disease. These disease-associated mutations (DAMs) are known to occur predominantly in positions essential to the structure and function of the proteins. Here, we examine how the relative perpetuation and conservation of amino acid positions modulate the genome-wide patterns of 8,627 human disease-associated mutations (DAMs) reported in 541 genes. We compare these patterns with 5,308 non-synonymous Single Nucleotide Polymorphisms (nSNPs) in 2,592 genes from primary SNP resources.

**Results:**

The abundance of DAMs shows a negative relationship with the evolutionary rate of the amino acid positions harboring them. An opposite trend describes the distribution of nSNPs. DAMs are also preferentially found in the amino acid positions that are retained (or present) in multiple vertebrate species, whereas the nSNPs are over-abundant in the positions that have been lost (or absent) in the non-human vertebrates. These observations are consistent with the effect of purifying selection on natural variation, which also explains the existence of lower minor nSNP allele frequencies at highly-conserved amino acid positions. The biochemical severity of the inter-specific amino acid changes is also modulated by natural selection, with the fast-evolving positions containing more radical amino acid differences among species. Similarly, DAMs associated with early-onset diseases are more radical than those associated with the late-onset diseases. A small fraction of DAMs (10%) overlap with the amino acid differences between species within the same position, but are biochemically the most conservative group of amino acid differences in our datasets. Overlapping DAMs are found disproportionately in fast-evolving amino acid positions, which, along with the conservative nature of the amino acid changes, may have allowed some of them to escape natural selection until compensatory changes occur.

**Conclusion:**

The consistency and predictability of genome-wide patterns of disease- associated and neutral amino acid variants reported here underscores the importance of the consideration of evolutionary rates of amino acid positions in clinical and population genetic analyses aimed at understanding the nature and fate of disease-associated and neutral population variation. Establishing such general patterns is an early step in efforts to diagnose the pathogenic potentials of novel amino acid mutations.

## Background

The association of mutations with specific human inherited diseases has been known for over five decades [1]. These mutations can be single nucleotide changes (point mutations), insertion or deletion of nucleotides (indels), or gross chromosomal rearrangements; furthermore, they may occur in protein-coding and in non-coding regulatory regions. Of all known gene lesions associated with disease, approximately half are point mutations that change the encoded amino acid [2]. Statistical analyses of these amino acid mutations are the most tractable, because their properties and tendencies can be predicted based on the long-term evolutionary history of their locations by comparative genomics [3-9].

Evolutionary analyses of disease-associated mutations (DAMs) have revealed a number of trends. They are over-abundant at positions that have remained unchanged in species that diverged hundreds of millions of years ago [4-6,8,9], and there is a general under-abundance of DAMs in positions that show any potential to change [3,5]. DAMs are more radical in terms of the differences in their biochemical properties from the normal amino acids, as compared to the differences observed between species [3,5,10]. Furthermore, only a very small fraction of known DAMs are identical to the inter-specific substitutions at the same positions [5,11-13]. In addition, the evolutionary history of amino acid positions and the long-term substitution patterns observed in the proteins have been employed with varying degrees of success in predicting the disease propensity of mutations [3,5,7,9,14-16].

However, many of the patterns mentioned above have been elucidated from the analysis of a limited number of proteins or mutations. With the recent expansion of genome and population variation data, it is now possible to establish molecular evolutionary anatomies of DAMs at a genome-scale and to use different measures of the intensity of natural selection at amino acid positions over the long term history of proteins. Although the significance of the biochemical severity of amino acid changes and their association with human diseases is well-appreciated, the possible relationship between the extent of biochemical dissimilarity of DAMs and the severity of human diseases (in terms of the time of onset of diseases) needs to be further explored. Similarly, the pattern of occurrence of non-synonymous polymorphisms (nSNPs) at sites evolving with vastly different intensities of natural selection are yet to be contrasted with those seen for DAMs.

Therefore, we undertook a genomic-scale analysis to elucidate the global evolutionary trends of rare Mendelian DAMs and nSNPs present in human proteins. We specifically examined the following questions: (1) What is the relative distribution of DAMs and nSNPs at positions that evolve with different rates? (2) Does the degree of retention of amino acid positions in non-human vertebrates show a relationship with the frequency of occurrence of DAMs and nSNPs? (3) How are the allele and genotype frequencies of nSNPs modulated by evolutionary variability of amino acid positions? (4) What is the relationship between the severity of inter-specific amino acid substitutions and the level of evolutionary conservation of positions harboring them? (5) What is the relationship between the biochemical severity of DAMs and the timing of the onset of different diseases? (6) To what extent does the evolutionary rate of a position explain the observed overlap between the inter-specific substitutions and DAMs? In order to answer these questions, we compared and contrasted available information on disease-associated amino acid mutation data, human population polymorphism data, and inter-specific amino acid difference data.

## Results

The evolutionary conservation of an amino acid position in a protein was measured in two ways. First, we estimated the rate at which amino acid substitutions have occurred at each position (Rate index). Secondly, we assessed the existence of a position in homologous proteins in species distantly and closely related to humans (Indel index). These two indices were estimated for human proteins where at least one DAM or nSNP has been reported in the public databanks (see Methods).

Both the Rate and Indel indices are estimated using multiple sequence alignments consisting of human proteins and their three closest homologs in the fully sequenced vertebrates (another mammal [*Mus musculus*], a bird [*Gallus gallus*], and a bony fish [*Takifugu rubripes*]). To estimate the rate index, we used a Maximum Likelihood (ML) procedure with a discrete-Gamma function to model differences in evolutionary rates among positions, while a JTT model was employed to account for differences in substitution probabilities between 20 amino acids [17,18]. The ML procedure indicated significant rate variation among positions (gamma shape parameter = 0.65) in all analyses, and predicted evolutionary rate at each position. They were arranged in eight rate categories (see Table 1). We also categorized positions using a simple Poisson model of amino acid substitution for estimating evolutionary rates, because measures of biochemical severity are known to be correlated with the JTT model. We have presented results from the JTT-model-based analyses only, because both methods produced extremely similar results.

Our Indel index is simply the number of times a human amino acid position was missing a homolog in the multiple sequence alignment with three other species. Its value ranged from zero to three, with the smallest number representing the position most retained (Table 2). A more sophisticated index with eight categories that weighted indels based on evolutionary closeness of the species produced results similar to those reported here (results not shown).

### Opposite patterns of occurrence of DAMs and nSNPs

**Figure 1:**
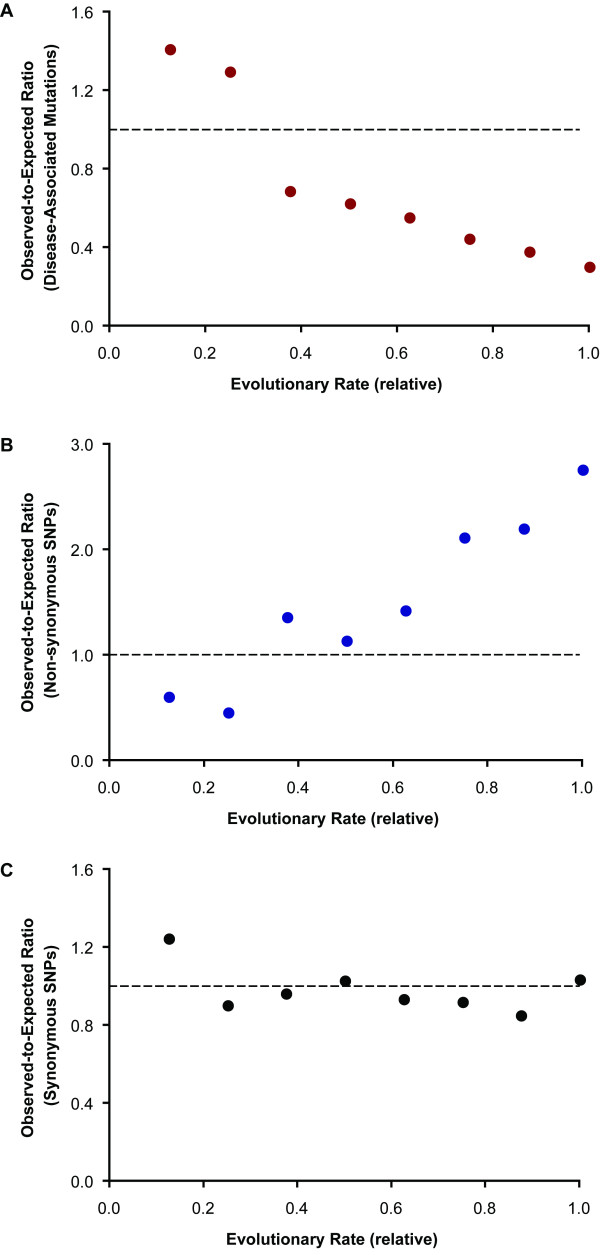
**Relationship of the observed-to-expected numbers of mutations in different evolutionary rate categories**. (A) Disease-associated mutations, DAMs (red). (B) Non-synonymous SNPs (blue), nSNPs. (C) Synonymous SNPs, sSNPs (black). The mid-values of the position rate categories are given in the X-axis (see also Table 1). The expected number of mutations at the amino acid positions belonging to a given rate index category, *i*, was computed as *E*_i _= (*n*_i_/*N*) × *M*, where *n*_i _is the number of amino acid positions belonging to the *i*th category, *N *is the total number of amino acid positions, and *M *is the total number of disease mutations (or SNPs) used in the analysis. The significance of the deviations of the observed values from the expected was evaluated by a χ^2 ^test. The significance of the deviations from the random expectations for DAMs was *P *<< 0.01 (χ^2 ^= 1318, *df *= 7), for non-synonymous SNPs was *P *<< 0.01 (χ^2 ^= 1586, *df *= 7); and for synonymous SNPs was *P *> 0.05 (χ^2 ^= 7.2, *df *= 7). The observed correlations are significant at a 1% level for DAMs (*R*^2 ^= 0.95) and nSNPs (*R*^2 ^= 0.92), and the correlation is insignificant for synonymous SNPs (*R*^2 ^= 0.21).

The relationship of the ratio of observed-to-expected counts of positions harboring DAMs and nSNPs in eight evolutionary rate classes is shown in Figure 1 for all positions that do not contain any insertion-deletions. An analysis of the distribution of 7,966 DAMs in 335,520 positions establishes their over-abundance in the slowest evolving positions (*P *<< 0.01; Figure 1A). The distribution of positions containing 4,022 nSNPs in 1,619,701 positions shows an opposite trend, as they are significantly under-abundant at the most highly conserved sites (*P *<< 0.01; Figure 1B). The observed tendencies may not be attributed to differences in mutational patterns among sites in different rate categories, because the observed-to-expected frequencies of 413 synonymous SNPs in the HapMap database (251,828 positions) show an expected random pattern (*P *> 0.05; Figure 1C).

**Figure 2:**
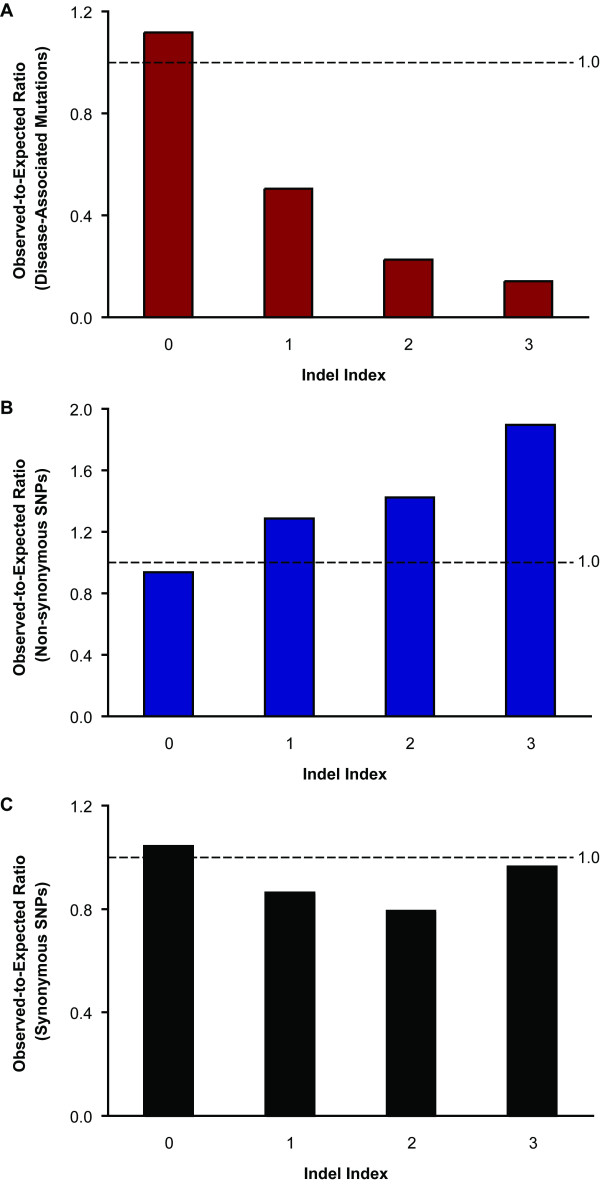
**Relationship of the observed-to-expected numbers of mutations in positions with different numbers of indels**. (A) Disease-associated mutations (red). (B) Non-synonymous SNPs (blue). (C) Synonymous SNPs (black). The indel index in the X-axis indicates the number of alignment gaps in the three nonhuman vertebrates included in the study. Specifically, the category 0 includes only the amino acid positions that have no indel in the multiple sequence alignments. Similarly 1, 2, and 3 includes the positions that have one, two, and three indels in the nonhuman vertebrate sequences with respect to the human sequence. Deviations from the random expectations are significant for DAMs at *P *< 0.01 (χ^2 ^= 599, *df *= 3), significant for non-synonymous SNPs at *P *< 0.01 (χ^2 ^= 143, *df *= 3), and not significant for synonymous SNPs (*P *> 0.05; χ^2 ^= 3.7, *df *= 3).

An analysis of all 8,627 DAMs in 436,360 positions clearly shows that positions that have indels in other species contain fewer than expected DAMs (*P *< 0.01; Figure 2A). In contrast, the distribution of 5,308 nSNPs in 2,180,746 positions reveals an excess of nSNPs at positions with many indels (*P *< 0.01; Figure 2B). Therefore, DAMs and nSNPs show exactly opposite trends. Again, this difference cannot be attributed to average differences in mutation rates, because positions with many indels do not contain an excess of synonymous SNPs as seen in an analysis of 528 sSNPs in 353,524 positions (*P *> 0.05; Figure 2C).

### Earlier onset diseases associate with more radical amino acid mutations

We examined the average of biochemical (Grantham) distances for DAMs, nSNPs, and amino acid differences observed between species [3,5,19]. The average Grantham distance for 7,966 DAMs is 92.1, which is more than 50% higher than that observed for 148,558 interspecific differences (66.6) and nSNPs (65.0) (Table 3). The similarity of the Grantham distance for between species "neutral" differences and within species "neutral" variations is remarkable and indicates that, on an average, the biochemical nature of polymorphisms within a population is a snap-shot of the differences that exist among species.

**Figure 3:**
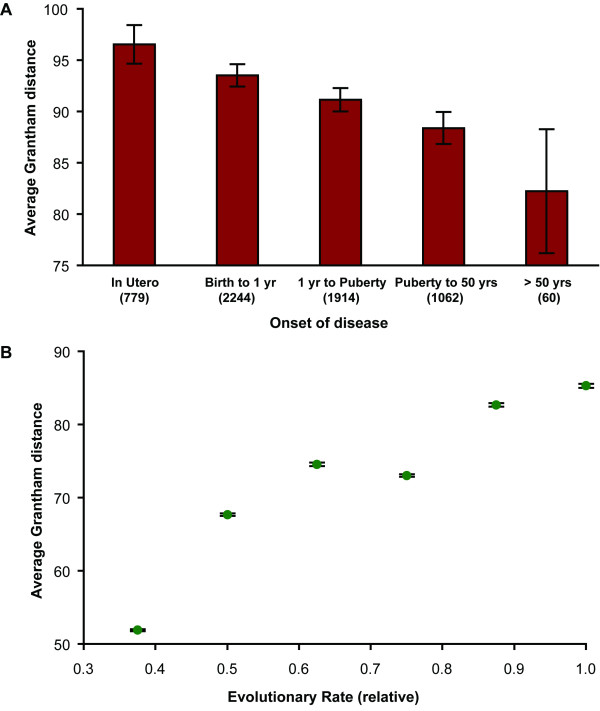
**Average biochemical distances between the amino acid residues found in the normal and disease humans and in other species**. (A) Relationship between the average Grantham distances estimated for the DAMs (red) and the time of onset of diseases (obtained from [20]). The numbers of genes are given in the parenthesis. Error bars show the standard error of the mean. (B) Correlation between the evolutionary rate and the average Grantham distance of the inter-specific amino acid substitutions (*R*^2 ^= 0.88, *P *< 0.01) (green). The average Grantham distance was estimated by including all the changes (with respect to the human proteins) that occurred in the amino acid positions of the non-human proteins belonging to various rate indices. Only six rate index categories were included, as the two categories include only the invariable amino acid positions. Error bars show the standard error of the mean.

DAMs can be further analyzed in the context of the timing of the diseases' onset. Diseases with onset at early stages of the human life cycle will be more harmful than those that are late-onset, because the former will affect fecundity more severely and will modulate the biochemical severity of DAMs. Indeed, the biochemical severity of mutations associated with diseases that manifest the earliest is 17% higher than the latest-onset diseases (*P *< 0.05). Even though the average differences between categories are small, there is a clear cut negative trend (Mann-Whitney U-test, *P *< 0.001; Figure 3A).

The average biochemical severity of DAMs was also analyzed in the context of the evolutionary rate of the positions, which do not show a significant monotonic trend (*P *= 0.21). In contrast, a positive relationship was observed between the evolutionary rate and the biochemical distances for inter-specific differences (Figure 3B), indicating that radical changes in highly variable positions are more tolerated than in positions with low evolutionary variability. This happens because more dissimilar amino acid changes will experience a higher intensity of purifying selection than the changes that involve highly similar amino acids.

## Discussion

We have observed opposite patterns of the distribution of disease-associated and non-synonymous variation in amino acid positions with different evolutionary rates, as well as indel propensities. These patterns are consistent with the predictions of the neutral theory of molecular evolution, because the purifying selection will eliminate mutations from functionally important positions more effectively. Both the rate index and indel index produce similar trends, because the natural selection will maintain the amino acid type and will retain the amino acid position among species.

It is important to note that we have considered different types of amino acids that are associated with disease mutations at different amino acid positions, and we have not considered the population frequency of DAMs. This is because allele frequencies for a vast majority of DAMs are either very small or are not known with great precision [20]. In order to make a direct comparison between DAMs and nSNPs, we repeated our analyses by using only lower-frequency HapMap nSNPs (allele frequency < 0.10), which confirmed the patterns reported in Figure 1B (*P *< 0.01).

Conversely, we looked for DAMs that occur in appreciable frequencies (> 10^-6^; [20]) and found them to be largely associated with late-onset diseases (post puberty). These mutations are not over-abundant at evolutionarily conserved positions (*P *= 0.7; 430 mutations), and they are biochemically less radical (Figure 3A). They are often associated with common diseases such as the hypertension, diabetes, and osteoporosis. The late onset of these diseases will result in a small affect on fecundity, which may explain why the positions harboring these DAMs do not have evolutionary imprints similar to those observed for other DAMs.

**Figure 4:**
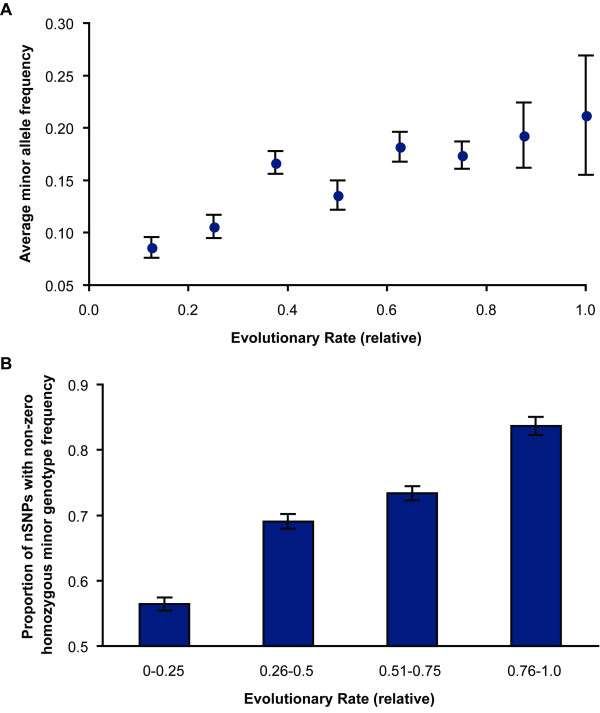
**Allele and genotype frequencies of non-synonymous SNPs at positions evolving with different evolutionary rates**. (A) Relationship of the average minor allele frequencies of the nSNPs with the evolutionary rate (*R*^2 ^= 0.85, *P *< 0.01) (blue). (B) The proportion of the nSNPs with a homozygous minor allele in at least one individual of the human population studied (blue). The rate index categories were merged into four bins, as mentioned in the Methods.

The invocation of natural selection to explain the observed distributions of DAMs and nSNPs makes a number of predictions that can be tested using the available information on nSNP allele frequencies from the HapMap data. As expected, minor allele frequencies of nSNPs are positively correlated with the evolutionary rate, because positions with higher long-term evolutionary rates are under lower purifying selection (*R*^2 ^= 0.85, *P *< 0.01; 935 nSNPs). The average minor allele frequency of the nSNPs at the highly conserved sites is less than half of that observed at the highly variable sites (Figure 4A).

The occurrence of homozygotes of minor alleles is also expected to correlate positively with evolutionary rates, because of the low minor allele frequencies and the heterozygous buffering effect when the minor alleles are deleterious. Therefore, we estimated the fraction of nSNPs for which the homozygous recessive genotypes occur with a non-zero frequency in the human populations examined. This proportion is the smallest for the highly conserved sites, and the highest for the most variable positions (Figure 4B).

**Figure 5:**
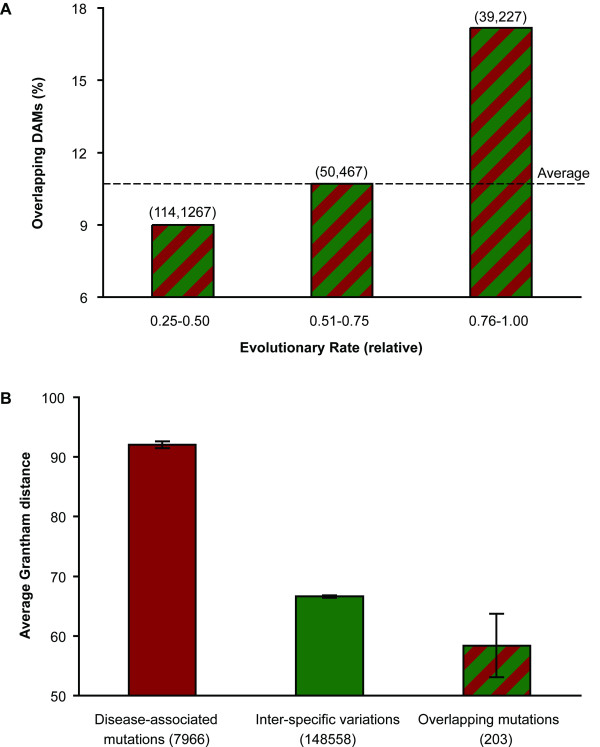
**Severity and frequency of overlapping DAMs**. (A) The proportion of DAMs that overlap with inter-specific variation at positions evolving with different evolutionary rates. Six rate index categories that show differences among species (Table 1) were pooled into three categories in order to make sample sizes large enough, because only 203 DAMs show overlap with inter-specific variations. The total number of DAMs and the overlapping DAMs are given in parenthesis. The hatched area indicates the fraction of DAMs (10.4%) that overlap with inter-specific fixed differences over all the variable amino acid positions (203/1961). (B) The average Grantham distance of DAMs (red), inter-specific variations (green), and the overlapping mutations (hatched). The numbers of mutations and differences are given in parenthesis. The error bars show standard error of the mean.

The neutral theory also predicts that DAMs at a given position will not be the same (amino acid) as fixed differences between species at that position, as long as the protein (and position) function remains unchanged over time, and the DAMs are significantly associated with the disease phenotype. This proves to be the case for an overwhelming majority of DAMs, as only 10.4% of them are identical to inter-specific variations. These proportions are similar to those reported earlier [11,13]. Overlapping DAMs are concentrated in the fast-evolving positions, as they contain twice as many of them as the slow-evolving positions (17% and 9%, respectively; *P *< 0.01). Therefore, the distribution of overlapping DAMs is not uniform (Figure 5A).

The overlap between the DAMs and inter-specific differences is often considered to be caused by the compensatory mutations, where the negative effects of the mutation(s) at one site of the same or different proteins compensates for the negative effects of the other mutation [11,13,21]. It is clear that such mutations need to escape natural selection for a period of time before the compensatory mutations can occur. This may only be possible for mutations that have very small negative fitness effects, in general. In terms of evolutionary rate, the overabundance of overlapping DAMs in fast-evolving positions is consistent with this expectation. The biochemical difference between the overlapping DAMs and the reference human amino acid is also consistent with this requirement, because the biochemical distance of overlapping DAMs is 38% lower than that observed for all other DAMs. In fact, overlapping DAMs are 14% more conservative than even the inter-specific variation (see also, [21,22]). Even though some of the overall patterns mentioned above are consistent with the compensatory mutation hypothesis, this is by no means the only or the primary explanation, because it is unclear what fraction of overlapping DAMs can be explained by compensatory mutation hypothesis. In the future, there will be a need to develop statistical approaches to examine contrasting hypothesis concerning the existence of overlapping DAMs, including the change in function of the protein or the position where the overlapping DAMs are seen.

## Conclusion

Our results emphasize the importance of the long-term evolutionary history of the amino acid positions and their influence in modulating the short-term history of the DAMs and nSNPs. Although our studies are restricted only to the protein-coding regions, the patterns reported here will hold true for DAMs and SNPs present in non-coding DNA containing conserved regulatory regions. Future studies using more species to examine the evolutionary conservation of amino acid positions could further improve the understanding of the mutations associated with human diseases and population variations. In particular, use of only the species that are closely related to humans, such as mammals, or, more specifically, primates, will prove to be more useful due to the similarity in their physiology and metabolism.

## Methods

### Proteomic data

Protein sequences of human (*Homo sapiens*) were obtained from GenBank build 34.1 [23], and mouse (*Mus musculus*), chicken (*Gallus gallus*) and fugu (*Takifugu rubripes*) were obtained from ensemble [24]. For each human gene, the putative orthologous gene, or closest sequence homolog, in the other three vertebrates were identified using a local BLASTP search with BLOSUM62 substitution matrix [25]. The threshold score (bit score *S *in BLASTP program) was set according to protein length (*L*): *S *= 150 for *L *≥ 170 amino acids, S = *L*-20 for 55 <*L *< 170 and S = 35 for *L *< 55 amino acids [26]. We used the reciprocal BLASTP search in which pairs of genes were considered orthologous only if they were mutually the best matches in their respective counterpart genomes [12]. We included only the human genes for which an orthologous counterpart was available in all the three vertebrate species. Each orthologous gene set was aligned with CLUSTAL-W using default settings [27]. Only the genes for which either a disease mutation or nSNP data was available were included for further analysis. We concatenated all the disease-associated genes and all the other genes (for which SNP data was available) separately, and all the sites containing any missing data or indels were excluded from the two concatenated alignments. The rate of evolution of individual sites was estimated by Maximum Likelihood analysis using PAML [17] with the JTT model of evolution. We used a discrete gamma model (with eight categories) to describe the distribution of evolutionary rates among sites. The complete amino acid alignments, including indels, were used for computing the indel index.

### Disease mutation and SNP data

We obtained 20,309 disease-associated mutations in 1,307 human genes from the HGMD database [28], and 29,856 non-synonymous SNPs in 11,753 known human genes were obtained from the HapMap project, Perlegen (March, 06), HGVBase (Human Genome Variation Database 17) and TSC (The SNP Consortium, 1). The HGVBase and TSC data were obtained through BioMart [24]. We excluded 1,215 mutations that were present in DAM as well as the SNP data, because public SNP resources may contain disease mutations even when "healthy" individuals are screened, especially when we consider DAMs for common, late-onset diseases. (We plan to conduct an analysis of these mutations in the future.) We have included only the disease mutation or nSNPs for which the wild-type amino acid and the amino acid in the human reference sequence (build 34) were the same. We were able to map 8,627 DAMs (in 541 genes) and 5,308 nSNPs (in 2,592 genes) to the concatenated alignments of the orthologous sequences using the positional information obtained from the respective databases. The allele and genotype frequencies (used in Figure 4) were available only for the HapMap data. The allele and genotype frequencies of synonymous and non-synonymous SNPs are available for four different ethnic populations (Utah Caucasian-Americans, Yoruba Africans, Hans Chinese, and Tokyo Japanese), and we took the average frequencies of the four populations. The HapMap SNPs with no variation in all the four populations (which was determined from the allele frequencies) were excluded in the analysis.

## Authors' contributions

The project was initially conceived by SK. SS undertook data acquisition, analysis, and tests as well as formulation of evolutionary hypotheses. The manuscript was written initially by SS, and then rewritten by SK and SS. All authors have read and approved the final manuscript.

## Figures and Tables

**Table 1:** Frequency of disease-associated and non-synonymous (nSNPs) mutations in different evolutionary rate categories

Rate index (estimated rate range)	Disease-associated genes (523)		Other genes (2264)	
	
	No. of positions	DAMs	No. of positions	nSNPs
0 (0.000–0.125)	90,971	3,047	339,162	510
1 (0.126–0.250)	95,991	2958	546,466	622
2 (0.251–0.375)	39,751	649	203,711	688
3 (0.376–0.500)	41,570	618	208,192	588
4 (0.501–0.625)	18,235	240	96,819	343
5 (0.626–0.750)	21,490	227	108,584	571
6 (0.751–0.875)	16,061	145	72,280	395
7 (0.876–1.000)	11,451	82	44,487	305
	
Total	335,520	7,966	1,619,701	4,022

NOTE: – The evolutionary rates of amino acid positions obtained from Maximum Likelihood analysis ranges from 0.32 to 3.4. For simplicity, we normalized each rate value by dividing by 3.4 in order to make the scale from 0.094–1.000. The amino acid positions were then grouped into eight categories based on their relative rate of evolution with an interval of 0.125.

**Table 2:** Frequency of disease-associated and non-synonymous (nSNP) mutations in different Indel index categories

Indel index^1^	Disease-associated genes (541)		Other genes (2592)	
	
	No. of positions	DAMs	No. of positions	nSNPs
0	360,070	7,966	1,781,946	4,022
1	58,452	585	312,656	971
2	15,356	69	69,298	238
3	2,482	7	16,846	77
	
Total	436,360	8,627	2,180,746	5,308

^1 ^-Number of nonhuman vertebrate proteins containing an alignment gap.

**Table 3:** The biochemical severity of disease-associated mutations, differences among species, and nonsynonymous mutations

Type	Number of observations	Grantham Distance	
		
		Average	Std. Error
Disease-associated	7,966	92.06	0.57
Nonsynonymous SNPs	4,022	64.95	0.65
Differences among species	148,558	66.61	0.19

^1 ^-Grantham measure [19] was used to quantify the biochemical distance between amino acids.
